# Psychometric Properties of the Dutch Depression Stigma Scale (DSS) and Associations with Personal and Perceived Stigma in a Depressed and Community Sample

**DOI:** 10.1371/journal.pone.0160740

**Published:** 2016-08-08

**Authors:** A. M. Boerema, K. van Zoonen, P. Cuijpers, C. J. M. Holtmaat, L. B. Mokkink, K. M. Griffiths, A. M. Kleiboer

**Affiliations:** 1 Department of Clinical Neuro and Developmental Psychology, Section clinical psychology, Faculty of Behavioural and Movement Sciences, Vrije Universiteit, Amsterdam, the Netherlands; 2 EMGO+ institute for Health Care and Research, Vrije Universiteit Medical Centre, Amsterdam, the Netherlands; 3 Statistics Netherlands (CBS), Den Haag, the Netherlands; 4 Department of Epidemiology and Biostatistics, VU University Medical Center, Amsterdam, the Netherlands; 5 National Institute for Mental Health Research, The Australian National University, Acton, Canberra, Australia; Iranian Institute for Health Sciences Research, ISLAMIC REPUBLIC OF IRAN

## Abstract

**Background:**

Research on depression stigma is needed to gain more insight into the underlying construct and to reduce the level of stigma in the community. However, few validated measurements of depression stigma are available in the Netherlands. Therefore, this study first sought to examine the psychometric properties of the Dutch translation of the Depression Stigma Scale (DSS). Second, we examined which demographic (gender, age, education, partner status) and other variables (anxiety and knowledge of depression) are associated with personal and perceived stigma within these samples.

**Methods:**

The study population consisted of an adult convenience sample (*n* = 253) (study 1) and a community adult sample with elevated depressive symptoms (*n* = 264) (study 2). Factor structure, internal consistency, and validity were assessed. The associations between stigma, demographic variables and anxiety level were examined with regression analyses.

**Results:**

Confirmatory factor analysis supported the validity and internal consistency of the DSS personal stigma scale. Internal consistency was sufficient (Cronbach’s alpha = .70 (study 1) and .77 (study 2)). The results regarding the perceived stigma scale revealed no clear factor structure. Regression analyses showed that personal stigma was higher in younger people, those with no experience with depression, and those with lower education.

**Conclusions:**

This study established the validity and internal consistency of the DSS personal scale in the Netherlands, in a community sample and in people with elevated depressive symptoms. However, additional research is needed to examine the factor structure of the DSS perceived scale and its use in other samples.

## Introduction

Depression is a common mental disorder [1] and is associated with significant personal and social burden [2, 3] and enormous economic costs [4]. In the Netherlands, 6.1% of the adult population suffer from a mood disorder annually [5]. In addition to the difficulty of dealing with symptoms and disabilities that result from a depression, stigma associated with depression is a great concern as well [6]. Stigma refers to a ‘a mark of shame, disgrace or disapproval which results in an individual being rejected, discriminated against, and excluded from participating in a number of different areas of society’ [7]. Stigmatizing ideas about people with depression are common [6] and include the belief that people with depression are unpredictable or responsible for their own condition [8–10]. Depression stigma can result in poorer mental health of the individual with depression [11–15] and may negatively affect individuals’ willingness to seek help [8, 16, 17].

Stigma can manifest in different ways and three forms of stigma have been distinguished. *Personal stigma* refers to peoples’ own attitude towards people with depression [18]. *Perceived stigma* involves peoples’ beliefs about the negative attitudes of other people [18] and *self-stigma* refers to the internalization of negative attitudes of others towards the self (i.e., a person’s view of their own depression) [19]. Different factors may be associated with different forms of stigma. There is evidence that personal stigma is higher among men and people with less education [20, 21]. Furthermore, there are indications that a lower level of exposure to and knowledge of depression is associated with higher personal stigma [18, 21–23]. Higher perceived stigma has been reported among those with higher levels of symptom severity [21, 22, 24]. However, the findings for perceived stigma show greater inconsistency than for personal stigma [21], especially for demographic variables. For example, some studies have reported a higher level of perceived stigma among women [22] while other studies have found no association between gender and perceived stigma [21, 24].

In recent years, stigma has been recognized by policy makers and organizations as an important public health issue and several initiatives and interventions have been developed for reducing stigma in society [25, 26]. Although stigma interventions, such as educational and consumer contact, have been shown to result in small but significant reductions in personal stigma [18, 27], there is little evidence for the effectiveness of interventions in reducing perceived stigma [18, 27]. To optimize these interventions, more research is needed to examine the underlying construct of stigma. More research is also required to better understand stigma in people who suffer from depression, since most research on personal and perceived stigma is undertaken in community samples of people without a diagnosis of depressive disorder.

Currently, in the Netherlands, there is a lack of validated instruments for measuring stigma in depression. Therefore, it is important to investigate if already developed and tested questionnaires are suitable for use in the Netherlands. The Depression Stigma Scale (DSS) [18] was developed in Australia and has been used in a number of community samples. The scale distinguishes between personal and perceived stigma. The DSS is a brief questionnaire that is easy to use and is available in several countries. Different studies, in varied countries (e.g., Australia, Japan, Germany) and populations (e.g. national survey, local community and local community distressed subset) have demonstrated sufficient to good internal consistency and high test-retest reliability for the DSS [18, 21, 28–30]. Since the Netherlands and Australia are both high-income countries, with comparable mental health policies [31], and a health system in which the general practitioner serves as gatekeeper [32], the DSS may be an appropriate tool for measuring stigma in the Netherlands.

The aim of the present study was to examine the validity and internal consistency of the DSS in the Netherlands using two Dutch samples. In addition, we examined demographic variables and other predictors of personal and perceived stigma within these samples. This study established the validity and internal consistency of the DSS personal stigma scale in the Netherlands. However, additional research is needed to examine the validity of the DSS perceived stigma scale and its use in other samples.

## Method

### Participants

A convenience sample from the community (*N =* 253) (study 1) was recruited as part of a student project. Third year undergraduate students recruited participants within their own environment (e.g. family, friends, neighbors). The students were instructed to recruit people in different age categories, country of birth and gender, with the aim of collecting a sample of the community with a broad range of demographic attributes.

The sample for the second study was a subset of participants in a health survey that is conducted by the Municipal Health Services (GGD) in a random community sample every four years. This survey includes questions about physical health, life-style, environment, psychosocial health, and also the Kessler-10 (K-10), a screening questionnaire for psychological distress [33]. Participants who completed the Health Monitor in one of three regions in the Netherlands (Amsterdam, Zuid-Holland West, Zuid-Holland Zuid) in 2012, who scored above the cut-off score for elevated distress levels on the K-10 (>20), and who were 18 years of age or older were invited to participate in a study examining help-seeking behavior in people with elevated symptoms of depression from the Vrije Universiteit in Amsterdam [34]. The study of the Vrije Universiteit incorporated the DSS. People with insufficient understanding of the Dutch language were excluded. This paper reports the findings from the analyses of the data collected from the Vrije Universiteit study.

### Procedure

Study 1 was approved by the Ethical Committee (VCWE) of the Faculty of Psychology and Education at the Vrije Universiteit in Amsterdam. People who agreed to participate received an information letter and an informed consent form. They were asked to sign and return the informed consent form to the research team at the Vrije Universiteit. Next, participants were invited to complete an online questionnaire which took approximately 20 minutes.

Study 2 was approved by the medical ethical committee of the VUmc (nr 2011/394). Participants who scored above the threshold on the K-10 screening questionnaire, and who agreed that they could be approached for further study, were contacted by the GGD by post and received an information letter and an informed consent form. Participants were asked to sign and return the form to the research team at the Vrije Universiteit. Participants were then invited to participate in a telephone interview to determine their depression status (CIDI 2.1, not used in the present study) and to complete an online questionnaire.

### Instruments

#### Demographic variables

In study 1 and 2, information about age, gender, marital status and education level was collected.

#### Depression Stigma Scale (DSS)

The DSS consists of two subscales: the personal and perceived stigma scale [18]. The items are rated on a 5-point Likert scale ranging from strongly disagree (0) to strongly agree (4), with a total score for each subscale in the range of 0–36. A higher score indicates greater stigma. Sufficient to good internal consistency and high test-retest reliability for the DSS has been reported [18, 21, 29]. For the purposes of the current study, the DSS was translated into Dutch according to a multiple forward and backward procedure. Two independent researchers (KvZ and AK) translated the questions from English to Dutch. Differences in translation were discussed between researchers and a single version was agreed upon. This version was then translated back into English by a third researcher. That version was then compared to the original version of the DSS. Again, potential differences were discussed by two authors (KvZ and AK).

#### Hospital Anxiety and Depression Scale (HADS)

The HADS is a self-report screening scale which is designed to indicate the presence of a depressive or anxiety state [35]. The HADS consists of two subscales, one for depression and one for anxiety. Each subscale consists of 7-items measured on a 4-point likert scale with total scores ranging from 0–21. A higher score indicates more depressive or anxiety symptoms. The HADS has shown good internal consistency [35, 36]. The HADS depression subscale was only available in sample 1 (Cronbach’s α depression subscale = .70). The internal consistency of the HADS anxiety subscale in both studies was adequate (Study 1: Cronbach’s α anxiety subscale = .79; Study 2: Cronbach’s α = .83).

#### Self-reported experience with depression

Information about the respondent’s history of depression was asked using the following questions ‘Have you ever experienced depressive symptoms?’ and ‘Have you ever had treatment for depressive symptoms?’. Participants were also asked if they knew anyone with depressive symptoms and if that person had been treated for their depressive symptoms. This questionnaire was only available in study 1.

#### Social Distance Scale (SDS)

The SDS is a self-reported scale which measures the willingness to make contact with a depressed person described in a vignette [37]. Participants rated their willingness to: 1) move next door to the person in the vignette; 2) spend an evening socializing with the person; 3) make friends with the person; 4) work closely with the person; and 5) have the person marry someone in their family. The items are answered on a 4-point Likert scale (‘definitely willing’ to ‘definitely unwilling’). The total score is the mean rating across the 5 items with higher scores indicating less willingness to make contact. The SDS has been reported with good internal consistency [38]. The internal consistency in the present study was sufficient (Cronbach’s α = .72). This questionnaire was only available in study 1.

### Response rate

In study 1, information letters were sent to 314 people. All invited people returned their informed consent form and 253 participants completed the DSS questionnaire. This represents a response rate of 81%.

In study 2, information letters were sent to 1191 people. A total of 331 participants (Amsterdam, *n =* 140; Zuid-Holland West, *n =* 148; Zuid-Holland Zuid, *n* = 43) returned their informed consent, a response rate of 28%. 264 participants completed the DSS questionnaire, which is a response rate of 22%.

### Characteristics of the study sample

The sample in study 1 consisted of 145 women (56%) and the mean age was 34 years, ranging from 18 to 82. Thirty-six percent of the respondents reported that they had experienced a depression (*n* = 92) and 15% reported they had received treatment for depression at least once in their life (*n* = 38). The average score on the HADS anxiety subscale was 5.6 (SD *=* 3.67) and on the HADS depression subscale 3.76 (SD = 2.94) (Table 1).

**Table 1:** Population characteristics study 1 and 2.

	Community sample (*N =* 253)	Depressive symptoms (*N =* 264)
**Demographics**		
Gender, *n* (%), female	143 (57)	147 (56)
Age, mean (SD)	34 (14.6)	55 (17.6)
Education, *n* (%)		
low	30 (12)	89 (34)
middle	121 (48)	69 (26)
high	102 (40)	106 (40)
Partner status, yes *n* (%)	174 (68)	145 (50)
**Clinical characteristics**		
HADS anxiety, mean (SD)	5.57 (3.67)	7.45 (3.96)
HADS depression, mean (SD) ^a^	3.76 (2.94)	-
Ever had a depression? yes, *n* (%)^a^	92 (36)	-
Ever treated for depression? yes, *n* (%)^a^	38 (15)	-
**Stigma**		
Personal stigma, mean (SD)	12 (4.34)	13 (5.85)
Perceived stigma, mean (SD)	20 (4.69)	21 (5.71)
Social distance, willingness, *n* (%) ^a^^,^^b^		
Not willing to make contact	138 (55)	-
Willing to make contact	111 (45)	-
**Knowledge depression** ^a^		
Do you know someone with depression? yes, *n* (%)	189 (75)	-
Do you know someone who is treated for depression? *n* (%)	165 (65)	-

^a^ Information only available for study 1

^b^ n = 249

The sample in study 2 consisted of 147 women (56%) and the mean age was 55 years, ranging from 19 to 94. Just over one-third (36%) of the respondents met the criteria for MDD (*n* = 106). The mean of the HADS anxiety subscale was 7.5 (SD *=* 3.94) (Table 1).

### Statistical analyses

#### Psychometric characteristics of the stigma scales

MPlus version 7.3 was used to examine the psychometric properties of the DSS, using a Confirmatory Factor Analysis (CFA) [39]. Item responses were categorical (ordinal Likert data). Therefore we used the weighted least squares with adjustments for means and variances estimation (WLSMV), using polychoric correlation coefficients, which are considered robust with ordinal item responses. Model fit was examined using the Comparative Fit Index (CFI), Tucker-Lewis Index (TLI), Root Mean Square Error of Approximation (RMSEA) and Standardized Root Mean Square Residual (SRMR). The criteria for an acceptable or good model fit were: CFI and TLI values >.95, RMSEA values < .06 and SRMR values of < .08 [40]. The analyses were performed separately on the personal and perceived stigma subscales.

In the first instance the CFA was undertaken on sample 1. Since the initial CFA did not demonstrate a good model fit for this sample, an Exploratory Factor Analysis (EFA) was conducted to further test the data. This analysis used the WLSMV method, with oblique Geomin rotation. Number of factors were assessed with a scree plot and initial eigenvalues (>1). Individual items were included if their component loadings were .32 or higher. Thereafter, a second set of CFA analyses was conducted in sample 2 (*N =* 264) to test the structure identified by the EFA analyses.

After the above analyses, the internal consistency for each unidimensional (sub)scale was examined with Cronbach’s alpha. Alpha between 0.70 and 0.95 was considered appropriate [41]. Convergent validity was then assessed by testing hypotheses about expected correlations between the DSS personal stigma subscale and the SDS in sample 1. Based on earlier research findings we expected a strong correlation between the DSS and SDS (*r* = .51) (21). Divergent validity was assessed by comparing the correlation between the DSS personal stigma scale and the DSS perceived stigma scale and comparing the DSS perceived stigma scale with the SDS. Based on earlier research findings we expected a weak correlation (*r* = .12) [21]. Convergent validity was only assessed in study 1.

#### Predictors of stigma

A two-step hierarchical linear regression analysis with demographic variables and HADS anxiety score in the first step and other factors in the second step was performed to determine the association between the factors (gender, age, education level, and anxiety) and the dependent variable (personal or perceived stigma).

First, we conducted a series of univariate analyses for each predictor separately. Then we conducted a multivariable analysis in which all the determinants were included: gender (0 = male, 1 = female); age (centered); partner status (0 = no partner, 1 = partner); education (0 = low, 1 = high); ever had a depression (0 = yes, 1 = no); ever had someone with depression nearby (0 = yes 1 = no); anxiety (continuous scale). The assumptions of linear regression analyses (multicollinearity, linearity, normally distributed errors, Durbin Watson test (independent errors), homoscedasticity, standardized residuals) were tested and there was no indication of abnormality. The analyses were performed separately on the personal and perceived stigma scale and also separately on the two data samples.

## Results

### Depression stigma scale

In study 1 the average scores on the personal and perceived stigma scale were respectively 12 (SD *=* 4.34) and 20 (SD = 4.69). The average scores in study 2 were similar to study 1: personal stigma: *M =* 13 (SD *=* 5.85) and perceived stigma: *M =* 21 (SD = 5.27) (Table 1).

### Psychometric characteristics of the stigma scales

#### Results of the CFA analyses for the personal stigma scale (Sample 1, N = 253)

Initial CFA analysis, with all 9 items loading on the latent variable personal stigma, resulted in poor model fit (χ ^2^ = 20.21, p>.001; CFI = .74, TLI = .66, RMSEA = .11; SRMR = .07). The data showed that some items (item 2 & item 3; item 4 & item 6; item 8 & item 9) had high residual correlations, indicating that the assumption of local independence was violated.

#### Results EFA analyses Sample 1 (N = 253)

The results from the initial CFA analyses indicated that item 1 (‘People with depression could snap out of it if they wanted’) did not load on any factor (factor loading .18, R-square = .03). Consequently, we performed the EFA analysis without item 1. The scree plot and initial eigenvalues (2.96, 1.18, 1.16) indicated a 3 factor solution for the personal stigma scale (*Weak-not-sick-avoidance*: item 2,3,5,7 *Dangerous/unpredictable*: item 4 & 6 and *Discrimination*: 8 & 9). The results confirmed an acceptable model fit for the three-factor solution (χ ^2^ = 8.21, *df* = 7, *p* = .31) (Table 2). The factor loading matrices for the models are shown in Table 3.

**Table 2:** Model fit from exploratory factor analysis personal stigma scale, n = 253.

	χ ^2^	df	p
Model			
EFA model 1: 1-factor	194.54	20	< .001
EFA model 2: 2-factor	75.45	13	< .001
EFA model 3: 3-factor	8.21	7	.32

*Note* Item 1 was excluded in all models.

**Table 3:** Factor loadings from exploratory factor analysis personal stigma scale, n = 253.

	Model 1	Model 2		Model 3		
Items	Factor 1	Factor 1	Factor 2	Factor 1	Factor 2	Factor 3
2. Depression is a sign of personal weakness	.67	**.82**	-.004	.**83**	-.006	-.04
3. Depression is not a real medical illness	.55	**.64**	**-**.009	**.71**	-.10	-.01
4. People with depression are dangerous	.41	**.29**	.21	.12	.**51**	.01
5. It is best to avoid people with depression so that you don’t become depressed yourself	.55	**.51**	.13	**.49**	.12	.06
6. People with depression are unpredictable	.34	**.26**	.16	-.001	**.91**	-.20
7. If I had a depression I would not tell anyone	.47	**.45**	.12	**.43**	.11	.06
8. I would not employ someone if I knew they had been depressed	.65	-.01	**.82**	-.007	-.03	**.90**
9. I would not vote for a politician if I knew they had been depressed	.71	.11	**.74**	.15	.01	**.67**

#### Results CFA analyses (Sample 2, N = 264)

Next, we examined the three factor solution identified from the EFA using a CFA of the data from sample 2 (WLSMW method) without item 1. The results showed acceptable model fit for the CFI, TLI and WRMR indices for the derived model. Modification indices showed that allowing a residual covariance between DSS_2 (‘Depression is a sign of personal weakness) and DSS_3 (‘Depression is not a real medical illness’) resulted in a better fit (χ ^2^ = 28.75, *df* = 16, *p* = .03; CFI = .99; TLI = .98; RMSEA = .06;WRMR = .50) (Table 4).

**Table 4:** Model fit from confirmatory factor analysis personal stigma scale, n = 264.

	χ ^2^	df	p	CFI	TLI	RMSEA	WRMR
Model							
CFA model 1: 3-factor solution from EFA	65.03	17	< .001	.96	.93	.10	.79
CFA model 2: 3-factor solution from CFA with residual covariance	28.75	16	.03	.99	.98	.06	.50

*Note*. Model 1 = Model 3 from Exploratory Factor Analysis without item 1; Model 2 = residual covariance allowed between DSS_2 and DSS_3 without item 1

The results showed moderate to high correlation between the three factors (F2-F1 = .69; F3-F1 = .71; F2-F3 = .51). Therefore, we examined if a second order structure resulted in a better fit than the single-order CFA. The results of the second order CFA resulted in the same fit as the CFA model 1 (Table 4).

#### Internal Consistency

Cronbach’s alpha for the personal stigma scale in study 1 was respectively .67 including the non-suitable item and .70 without this item. Cronbach’s alpha for the personal stigma scale in study 2 was .77.

#### Results of the CFA analyses for the perceived stigma scale (Sample 1, N = 253)

Initial CFA analysis, with all 9 items loading on the latent variable perceived stigma, resulted in poor model fit (χ ^2^ = 694.16, p < .001; CFI = .76, TLI = .68, RMSEA = .22; WRMR = 2.57). The data showed that some items (item 11 & item 12; item 13 & item 15; item 17 & item 18) had high residual correlations, indicating that the assumption of local independence was violated.

#### Results EFA analyses (Sample 1, N = 253)

The scree plot and initial eigenvalues (3.56, 1.24, 1.21) indicated a 3 factor solution for the perceived stigma scale. The results confirmed an acceptable model fit for the three-factor solution (χ ^2^ = 10.62, *df* = 12, *p* = .56) (Table 5). The factor loading matrices for the three models are shown in Table 6.

**Table 5:** Model fit from exploratory factor analysis perceived stigma scale, n = 253.

Model	χ ^2^	df	p
EFA model 1: 1-factor	329.10	27	< .001
EFA model 2: 2-factor	175.48	19	< .001
EFA model 3: 3-factor	10.63	12	.56

**Table 6:** Factor loadings from exploratory factor analysis perceived stigma scale, n = 253.

	Model 1	Model 2		Model 3		
Items	Factor 1	Factor 1	Factor 2	Factor 1	Factor 2	Factor 3
10. Most people believe that people with depression could snap out of it if they wanted	.46	**.45**	.06	**.54**	-.06	.07
11. Most people believe that depression is a sign of personal weakness	.73	**.75**	.03	**.89**	-.004	-.003
12. Most people believe that depression is not a real medical illness	.62	**.65**	-.007	**.64**	.09	-.02
13. Most people believe that people with depression are dangerous	.62	**.67**	-.005	-.02	**.95**	.002
14. Most people believe that it is best to avoid people with depression so that you don’t become depressed yourself	.59	**.62**	.001	**.42**	.31	.005
15. Most people believe that people with depression are unpredictable	.63	**.69**	-.02	.15	**.69**	.00
16. Most people would not tell anyone if they had depression	.36	**.35**	.05	**.35**	.04	.05
17. Most people would not employ someone they knew had been depressed	.69	-.001	**1.42**	-.01	-.003	**1.22**
18. Most people would not vote for a politician they knew had been depressed	.58	.20	**.42**	.03	.13	**.52**

Item 17 (‘Most people would not employ someone they knew had been depressed’) had a factor loading >1 (1.22) (Table 6) and a negative residual (-.48), indicating the extraction of too many factors or an inadmissible solution [39]. However, the two-factor solution, including item 17, resulted in a poor model (Table 5; model 2). Therefore, item 17 was excluded. The EFA without item 17, resulted in a two-factor solution for the perceived stigma scale. However, in this solution, item 18 did not load on any component. Therefore, we performed the EFA analyses without both items. The EFA without item 17 and 18, resulted in a two-factor solution for the perceived stigma scale (Table 7 and Table 8).

**Table 7:** Model fit from exploratory factor analysis perceived stigma scale, without item 17 and 18, n = 253.

Model	χ ^2^	df	p
EFA model 4: 1-factor without item 17&18	164.65	14	< .001
EFA model 5:2-factor without item 17&18	9.48	8	.30

**Table 8:** Factor loadings from exploratory factor analysis perceived stigma scale, without item 17 and 18, n = 253.

EFA Model 5: 2-factor without item 17 &18	Factor 1	Factor 2
10. Most people believe that people with depression could snap out of it if they wanted	**.56**	-.05
11. Most people believe that depression is a sign of personal weakness	**.89**	-.004
12. Most people believe that depression is not a real medical illness	**.64**	.10
13. Most people believe that people with depression are dangerous	-.006	**.93**
14. Most people believe that it is best to avoid people with depression so that you don’t become depressed yourself	**.42**	.31
15. Most people believe that people with depression are unpredictable	.14	**.70**
16. Most people would not tell anyone if they had depression	**.37**	.05

*Note*. Factor scores represent the scores of model 5 in Table 7.

#### Results CFA analyses (Sample 2, N = 264)

Next, we examined the two factor solution in sample 2 using a CFA (WLSMW method) without the items 17 and 18. The results showed poor model fit for the CFI, TLI and WRMR indices for the derived model. (Table 9).

**Table 9:** Model fit from confirmatory factor analysis, n = 264.

Model	χ ^2^	df	p	CFI	TLI	RMSEA	WRMR
CFA model 1: 2-factor solution from EFA (without the items 17 and 18)	114.71	13	< .001	.92	.82	.17	1.06

#### Internal Consistency

Research showed that reporting a Cronbach’s alpha is only valuable when a scale is unidimensional [42]. Since we could not confirm the factor structure of the DSS perceived stigma subscale in the present study we decided not to report Cronbach’s alpha.

#### Convergent validity study 1

Concerning convergent validity, there was a significant association between the DSS personal stigma score and the SDS social distance score although the effect was small (*r* (247) = .28. *p* < .001).

#### Divergent validity and predictors for perceived stigma study 1&2

As already mentioned, we did not found a clear factor structure for the perceived stigma scale in the present study. Consequently, correlations with other questionnaires and regression coefficients may be more difficult to interpret. For this reason we decided not to examine divergent validity and predictors of the DSS perceived stigma subscale.

### Predictors of personal stigma

#### Study 1 Community sample (N = 253)

Univariate analyses showed that gender, age, education and experience with depression (self and surrounding) were associated with personal stigma. Being female, older and having a higher education level were associated with less personal stigma. No experience with depression was associated with higher personal stigma. Multivariate analyses showed that personal stigma was lower in people who were older. Personal stigma was higher in people without experience of depression in their environment (R^2^ = .10) (Table 10).

**Table 10:** Community sample (study 1): Univariate and Multivariate linear regression analyses for determinants predicting personal stigma.

	Univariate analyses			Multivariate analyses		
	Personal stigma			Personal stigma		
	B	SE B	*β*	B	SE B	*β*
**Demographic characteristics**						
Gender, female	-1.11	0.55	-0.13*	-0.64	0.56	-0.08
Age (centered)	-0.05	0.02	-0.15*	-0.04	0.02	-0.15*
Partner status, *yes*	-0.08	0.59	-0.009	-0.02	0.60	-0.002
Education, *high*	-1.21	0.55	-0.14*	-0.74	0.56	-0.09
HADS anxiety	-0.15	0.07	-0.13	-0.10	0.08	-0.08
**Other variables**						
Ever had depression, *no*				0.65	0.59	0.07
Ever had someone in their environment with depression, *no*				1.71	0.65	0.17*
***R***^**2**^ **(*R***^**2**^ **adjusted)**				.10 (.07)		
***R***^**2**^ **change**				0.04		
***F* change**				4.58		
**Sig F change**				0.01		

*p ≤ 0.05

#### Study 2 Sample with elevated depressive symptoms (N = 264)

Univariate analyses showed that personal stigma was associated with greater age and educational level. Older age was associated with more personal stigma. A higher educational level was associated with less personal stigma. Multivariate analyses showed that personal stigma was lower for people with high education compared with the reference group (low education) (R^2^ = .10) (Table 11).

**Table 11:** General population with depressive symptoms (study 2): Univariate and Multivariate linear regression analyses for determinants predicting personal stigma.

	Univariate analyses			Multivariate analyses		
	Personal stigma			Personal stigma		
	B	SE B	*β*	B	SE B	*β*
**Demographic characteristics**						
Gender, female	-1.14	0.73	-0.10	-0.85	0.73	-0.07
Age (centered)	0.06	0.02	0.18*	0.04	0.02	0.10
Partner status, *yes*	0.99	0.72	0.08	0.94	0.72	0.08
Education, *high*	-3.18	0.72	-0.27*	-2.89	0.74	-0.24*
HADS anxiety	-0.07	0.09	-0.05	-0.07	0.09	-0.05
**Other variables**						
***R***^**2**^ **(*R***^**2**^ **adjusted)**				.10 (.08)		
***R***^**2**^ **change**				0.10		
***F* change**				5.56		
**Sig F change**				0.000		

*p ≤ 0.05 ** *p ≤*0.001

## Discussion

The aim of this paper was to assess the psychometric properties of the Dutch translation of the DSS in two different samples: a convenience sample from the community and a sample with elevated levels of depressive symptoms. Furthermore, we examined predictors of depression stigma in each of the populations.

The initial CFA analyses did not confirm an unidimensional construct for the personal and perceived stigma scale [18, 21]. Therefore, we decided to perform an EFA to examine the underlying structure of the personal and perceived stigma scales. Since the DSS is used in many countries and different samples [21, 28] we decided to examine the generalizability in the two samples by performing an EFA in sample 1 and a CFA in sample 2. The EFA showed a 3-factor solution for the personal stigma scale (*Weak-not-sick-avoidance*: item 2,3,5,7 *Dangerous/unpredictable*: item 4 & 6 and *Discrimination*: 8 & 9). This is in line with previous research findings that showed that personal and perceived stigma are not unitary dimensions [43, 44]. The CFA analyses confirmed the 3 factor solution from the EFA. Furthermore, the CFA analyses showed a second order model (‘personal stigma’), indicating that the three factors are part of one overarching construct.

For the perceived stigma scale, we could not confirm the factor structure in the present study. The EFA analyses revealed a 3- factor solution for the perceived stigma scale (comparable to the factors of the personal stigma scale). However, item 17 (‘Most people would not employ someone they knew had been depressed”) was problematic (factor loading >1 and negative variances). By removing this item, item 18 (‘Most people would not vote for a politician they knew had been depressed’) did not load on any component. The present study findings suggests that these proxy social distance items measure a distinct factor which requires further research by, for example, identifying additional items that measure the same construct. However, other research findings have reported that items 17 & 18 load on the ‘Dangerous/Unpredictable’ factor [43, 44], suggesting that these items do not form a separate construct (in the present study ‘Discrimination’). A possible explanation for the failure of the CFA analyses to confirm the EFA findings for perceived stigma may lie in the different methods and samples used in the present study. For example, we used a sample with people who reported elevated symptoms of depression and a relatively healthy sample. Possibly, perceived stigma works differently for these two groups. However, other studies have demonstrated similar factor structures for the perceived stigma scale in different populations [21, 43, 44] and the structure for the personal stigma construct did not differ in the current samples.

The perceived stigma scale subscale scores are similar in both samples (M = 20, SD = 4.69); M = 21, SD = 5.71 respectively), and compared to the scores of the personal stigma scale (M = 12, SD = 4.34), M = 13, SD = 5.85, respectively) relatively high. This is in line with previous findings on the DSS [23, 28, 30]. Possibly, the scores on the perceived stigma scale reflect a social desirability bias [23, 30] where the perceived stigma scale scores may be a more accurate representation of stigma. Alternatively, people may overestimate perceived stigma in society [21, 23]. Possibly, these value judgements within the perceived stigma scale makes it more difficult to define a clear construct. However, the items of the personal stigma scale are almost similar, and the personal stigma construct works relatively well. It is clear that more research is needed to examine the factor structure of the perceived stigma scale in different populations. This factor may be improved for future research.

Item 1 in study 1 (‘‘People could snap out of depression if they wanted to”) did not load on any component in the community sample. One reason might be that the item’s translation was not adequate. Whereas ‘snapping out of something’ is a common expression in English there is no comparable expression in Dutch. Reframing the sentence in another study might lead to a better fit. However, the item’s performance was adequate in the sample with elevated depressive symptoms and the equivalent item performed well on the perceived stigma scale in both samples. The Cronbach’ s alphas of the DSS personal subscale was fair to good in both samples supporting the internal consistency. However, we did not find convincing support for the convergent validity of the DSS. The correlation between the DSS personal stigma scale and the social distance scale was weaker (*r =* .28) as expected (*r =* .53) [21]. In addition, we were not able to examine the divergent validity in the present study. Additional research is necessary to examine the convergent and divergent validity of the DSS in the Netherlands.

In summary, based on the findings in the present study, more research is needed to examine the psychometric properties of the Depression Stigma Scale (DSS) in the Netherlands. Regarding the personal stigma scale, our research findings are similar to other studies [43, 44]. Furthermore, convergent validity and internal consistency of the personal stigma scale seem sufficient. Based on the research findings in the present study, we could not confirm the factor structure of the perceived stigma scale. This factor may be improved for future research. However, a clear factor structure for the perceived stigma scale has been found in other studies [43, 44]. Furthermore, earlier research findings showed sufficient validity and test-reliability for the DSS perceived stigma scale (27). Possibly, the factor structure of the perceived stigma subscale could be confirmed in other Dutch samples. More research is necessary to replicate these findings.

Within the samples, there was a large diversity in predictors for personal stigma. In study 1 higher personal stigma was associated with people who were younger and those who did not know someone with depression, which is in line with previous findings [20, 21, 45, 46]. In study 2 personal stigma was lower in the high education group [20, 21]. Although the findings are broadly consistent with previous findings, we found an effect of gender on personal stigma only in the univariate analyses in study 1.This contrasts with previous studies that have reported that gender is associated with personal stigma [21]. The effects found in the multivariate analyses are in the same direction as those reported in other research (being male is associated with higher personal stigma), but were not statistically significant. A possible explanation might be that the study lacked power given that the majority of people in this sample were female and the effects were rather small. Alternatively, it is possible that there are true differences across countries in gender effects for stigma.

A comparison of the results of the samples showed that these were not consistent. The effect of education for personal stigma was only apparent in study 2, while the effect of age for personal stigma was only apparent in study 1. One possible explanation for the different pattern of findings in the two studies is that they are explained, at least in part, by differing population characteristics of the two samples. The respondents in study 1 were substantially younger (*M =* 34; *SD* = 14.56) than those in study 2 (*M* = 55; *SD* = 17.8). Furthermore, unlike the respondents in study 1, those in study 2 were selected for the presence of subclinical depressive symptoms. It may be noted that findings from previous research on the predictors of personal stigma have shown inconsistencies across studies. For example, some findings have reported that older age was associated with more personal stigma [21, 28, 47] while others have reported no effect of age [22]. These findings raise the possibility that predictors for personal stigma are dependent on which kind of sample is used in the analyses.

The results of the present study suggest that it is important to articulate the specific goals of anti-stigma campaigns. If the goal of the campaign is to increase awareness of stigma in members of the community (such as people in study 1), the interventions might target members of the community who are younger, less educated and people with no experience with depression in their environment. However, if the goal of a campaign is to reduce personal stigma among people who have elevated depressive symptoms we need to target women and people with a lower education level. Further research is needed to replicate these findings, since Griffiths and colleagues [21] have reported similar predictors for personal stigma in different samples (national household survey, local community and local community distressed subset). Moreover, the effects in the present study are small, which makes it difficult to reach persuasive conclusions.

There are several limitations that need to be considered when interpreting the results. First, the variance explained by the models was relatively low compared to other studies [21] and the effects were small. Further, we centered the age variable in the analyses due to the different distribution of age between the samples (Study 1: *M =* 34, SD = 14.6; Study 2: *M =* 55, SD = 17.6). By centering the age variable we were able to compare the results between the two study samples. However, the interpretation of these variables is more difficult. In addition, the design was cross-sectional so this study provides no evidence for causal relationships. Another limitation of the study was the use of a self-reported screening tool (HADS) to determine depression status in study 1. This may have led to under reporting of depression due to social desirability factors [28, 48]. Furthermore, some findings suggests that screening instruments are subject to gender bias [49, 50]. The use of a clinical diagnostic instrument, such as the CIDI 2.1 in study 2,may have been more appropriate. Unfortunately, it was not possible to assess depression status with the CIDI 2.1 in study 1 for pragmatic reasons. Further research should focus on designing a comprehensive study, employing comparable measurements for different age samples. In addition, it may be valuable to recruit a healthy and clinical sample within the same study, in order to examine convergent and divergent validity more thoroughly, by comparing these two groups.

## Conclusion

A validated instrument is of great importance in investigating stigma. Findings of the EFA, CFA and internal consistency support the use of the DSS personal stigma scale in the Netherlands. However, additional examination of the factor structure of the DSS perceived stigma scale and convergent and divergent validity of the DSS is necessary. This factor may be improved for future research. Since the DSS is already used in many other countries, comparisons can be made between countries. This in turn may facilitate collaborations between countries, provide further insight into stigma constructs and deliver useful information on targeting the right people for lowering stigma by means of public campaigns and interventions around the world.

## Supporting Information

S1 Dataset Dataset study 1. (SAV)

S2 Dataset Dataset study 2. (SAV)
